# Chronic alcohol consumption decreases brown adipose tissue mass and disrupts thermoregulation: a possible role for altered retinoid signaling

**DOI:** 10.1038/srep43474

**Published:** 2017-03-06

**Authors:** William S. Blaner, Madeleine A. Gao, Hongfeng Jiang, Timothy R. A. Dalmer, Xueyuan J. Hu, Henry N. Ginsberg, Robin D. Clugston

**Affiliations:** 1 Department of Medicine, Columbia University, New York, NY, USA; 2 Department of Physiology, University of Alberta, Edmonton, Alberta, Canada

## Abstract

Retinoic acid, an active metabolite of dietary vitamin A, acts as a ligand for nuclear receptor transcription factors with more than 500 known target genes. It is becoming increasingly clear that alcohol has a significant impact on cellular retinoic acid metabolism, with resultant effects on its function. Here, we test the hypothesis that chronic alcohol consumption impairs retinoic acid signaling in brown adipose tissue (BAT), leading to impaired BAT function and thermoregulation. All studies were conducted in age-matched, male mice consuming alcohol-containing liquid diets. Alcohol’s effect on BAT was assessed by histology, qPCR, HPLC, LC/MS and measures of core body temperature. Our data show that chronic alcohol consumption decreases BAT mass, with a resultant effect on thermoregulation. Follow-up mechanistic studies reveal a decreased triglyceride content in BAT, as well as impaired retinoic acid homeostasis, associated with decreased BAT levels of retinoic acid in alcohol-consuming mice. Our work highlights a hitherto uncharacterized effect of alcohol on BAT function, with possible implications for thermoregulation and energy metabolism in drinkers. Our data indicate that alcohol’s effects on brown adipose tissue may be mediated through altered retinoic acid signaling.

Our work is primarily focused on the effect of chronic alcohol consumption on hepatic vitamin A homeostasis and alcoholic liver disease^1^,^2^,^3^. Vitamin A is an essential dietary micronutrient that is important in many cellular processes including cell differentiation, proliferation and apoptosis. While visual phototransduction requires the vitamin A metabolite 11-*cis* retinal, the majority of the other physiological functions of vitamin A are mediated by all-*trans* retinoic acid. Simply referred to as retinoic acid in this text, this small molecule is a ligand for nuclear transcription factors (retinoic acid receptors), and has been linked to controlling the expression levels of more than 500 genes^4^. During our studies into the role of retinoic acid signaling in the pathogenesis of alcoholic liver disease, we observed that brown adipose tissue (BAT) weight was decreased in alcohol-fed mice, leading us to further study this phenomenon.

In 2009, three back-to-back papers published in the *New England Journal of Medicine* reported the presence of metabolically active BAT in adult humans^5^,^6^,^7^. This work triggered a surge of interest in BAT physiology, because of its significance with respect to energy metabolism and therapeutic implications for the management of diabetes and obesity^8^. Unlike white adipose tissue (WAT), which is optimized for energy storage, BAT is optimized for energy disposal^9^. This is achieved by the uncoupling of fatty acid oxidation in the mitochondria, leading to the generation of heat instead of ATP, an effect mediated by uncoupling protein 1 (UCP1)^10^. Interestingly, it has been suggested that UCP1 expression is retinoic acid-responsive, linking BAT function with tissue vitamin A homeostasis^11^. Furthermore, there is an older literature reporting altered BAT homeostasis in response to alcohol exposure in rodents. However, this so-called *hypothermic* effect of alcohol has primarily been studied in response to acute alcohol exposure^12^,^13^. A limited number of chronic studies have reported decreased BAT weight in response to chronic alcohol exposure, but this phenomenon has not been rigorously studied^14^,^15^,^16^. The current work tests the hypothesis that chronic alcohol consumption impairs retinoic acid signaling in BAT, leading to impaired BAT function and thermoregulation. This hypothesis is based on three pieces of information: (1) retinoic acid’s putative role in controlling UCP1 expression and BAT function; (2) altered BAT homeostasis following acute and chronic alcohol exposure; and (3) our preliminary data demonstrating that chronic alcohol consumption decreases BAT weight in mice. The following work includes a systematic study of alcohol’s effect on BAT in mice consuming alcohol. Based on our results, we conclude that chronic alcohol consumption impairs BAT homeostasis, an effect which may be mediated by altered retinoic acid signaling and impaired lipid metabolism. Our work highlights the effects of chronic alcohol consumption on BAT, as well as the potential importance of retinoic acid signaling in the maintenance of normal BAT function.

## Results

### Chronic alcohol consumption decreases BAT weight

**Figure 1:**
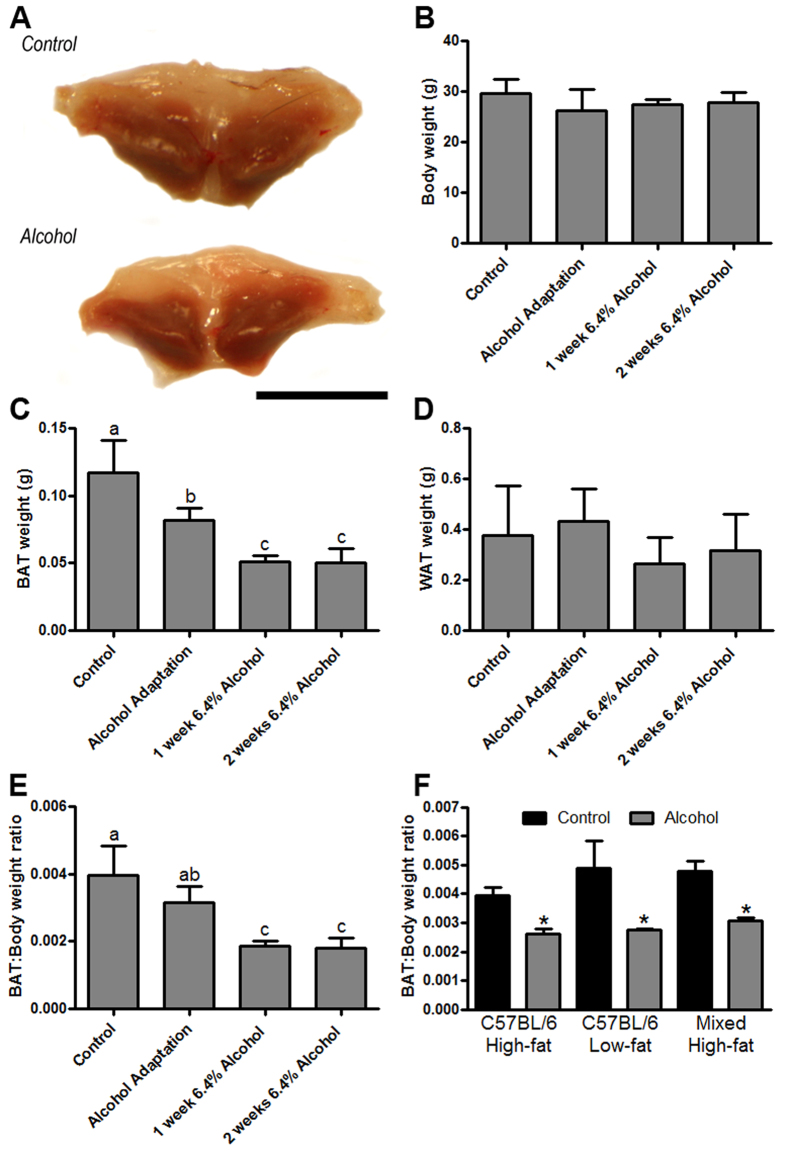
Chronic alcohol consumption decreases BAT mass. The dissected intrascapular BAT depot of alcohol fed mice is visibly smaller (**A**). Body weight was not changed in mice chronically consuming alcohol (**B**); however, BAT weight was significantly decreased throughout the alcohol feeding period, which was verified by analyzing the BAT:Body weight ratio (**E**). There was no significant change in the weight of the epididymal white adipose tissue (WAT) depot weight (**D**). Follow-up studies show that alcohol’s ability to decrease BAT mass occurs independently of dietary fat content, or genetic background (**F**). (**B–E**) analyzed by one-way ANOVA; bars with different letters are significantly different; p < 0.05. (**F**) analyzed by Student’s t-test; *p < 0.05. Sample size: (**B–E**), control n = 12, all alcohol groups n = 6; (**F**) all groups n = 5–6.

During our alcohol feeding studies we observed an apparent decrease in the size of the intrascapular BAT depot from alcohol-fed mice (Fig. 1A). Systematic follow-up analyses revealed that there was no significant difference in the body weight of alcohol consuming mice versus pair-fed control mice (Fig. 1B), yet the weight of the intrascapular BAT depot was markedly decreased in response to alcohol (Fig. 1C), with no significant change in WAT weight (Fig. 1D), or liver weight (control = 1.32 ± 0.2 g [n = 12] vs. alcohol = 1.33 ± 0.2 g [n = 5]). When BAT weight was expressed as a ratio of body weight, an alcohol-induced decrease in BAT was also observed (Fig. 1E). In order to control for the role of diet and genetic background in this observation, we measured BAT weight in several follow-up experiments. These data show that alcohol decreases BAT weight in C57BL/6 mice fed high-fat and low-fat formulations of the Lieber-DeCarli liquid diet, as well as in mixed background mice fed a high-fat liquid diet (Fig. 1F), compared to mice fed control alcohol-free liquid diets.

### Alcohol-fed mice have altered thermoregulation

**Figure 2:**
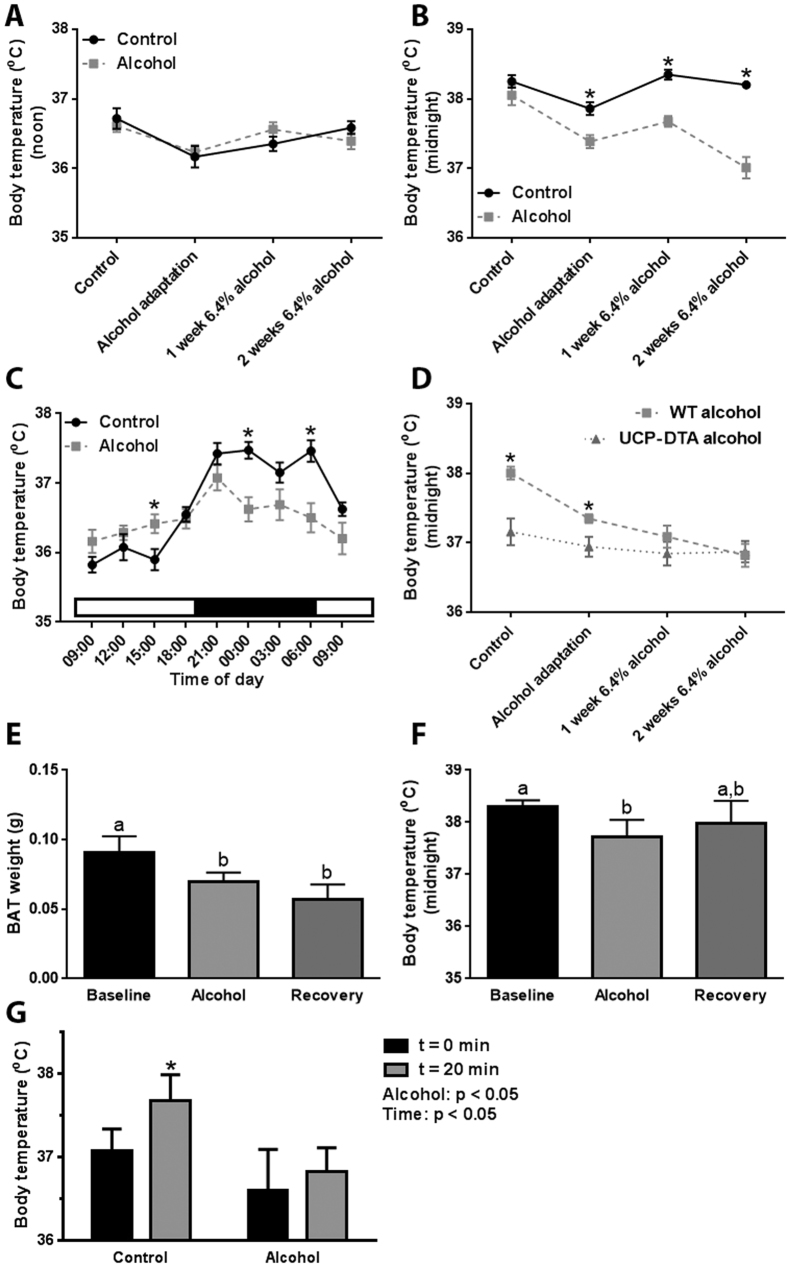
Chronic alcohol consumption is associated with altered thermoregulation. Core body temperature in alcohol consuming mice was not changed when measured at noon (**A**), but was significantly decreased when measured at midnight (**B**). Alcohol consumption was associated with an altered diurnal rhythm in core body temperature, characterized by decreased levels during the dark phase (**C**). Core body temperature is lower at baseline in UCP-DTA, but does not decrease in response to chronic alcohol consumption (**D**). Following a one month alcohol-free recovery period, BAT weight remained significantly decreased compared to control animals (**E**), and body temperature had not returned to baseline levels (**F**). In response to handling, control mice have a significant increase in core body temperature, but this effect is blunted in alcohol-fed mice (**G**). **(A-D)** analyzed by Student’s t-test; *p < 0.05; (**E,F**) analyzed by one-way ANOVA; columns with different letters are significantly different (p < 0.05); (**F**) analyzed by two-way ANOVA; *p < 0.05 vs. time = 0 min within groups. Sample size: (**A,B**) control and alcohol n = 12; (**C**) control and alcohol n = 8; (**D**) WT control = 12, WT alcohol = 18–22, and UCP-DTA alcohol n = 7; (**E,F**) all groups n = 5; (**E**) all groups n = 7.

Following the observation that alcohol decreases BAT weight, we set out to determine its effect on thermoregulation. When measured at noon, core body temperature was not significantly different between control and alcohol-fed mice (Fig. 2A); however, alcohol consumption was associated with a significant decrease in body temperature measured during the dark phase, paralleling the decrease in BAT weight (Fig. 2B). When body temperature was monitored throughout a 24-h period, we observed the expected diurnal pattern in control mice, with a lower temperature during the day and a higher temperature during the night (Fig. 2C). Consistent with the decrease in BAT weight, this diurnal pattern of body temperature regulation was significantly disrupted in alcohol-fed mice (Fig. 2C).

Our data revealed an association between decreasing BAT weight and nighttime body temperature; however, a direct cause and effect relationship could not be established. In order to determine if the alcohol-induced decrease in BAT weight was directly linked to disrupted thermoregulation, we conducted an alcohol feeding study in transgenic mice expressing diphtheria toxin A in UCP1-expressing cells (UCP-DTA mice), which have no BAT^17^. In addition to clarifying the link between decreased BAT weight and altered thermoregulation, this experiment provided an important control, addressing the possible acute effects on alcohol on BAT function, as well as any possible effects on the diurnal circadian body temperature rhythm. At baseline, UCP-DTA mice had significantly lower body temperatures than wild-type (WT) mice; however, when these mice were fed alcohol they did not experience a further decrease in body temperature (Fig. 2D), suggesting that the decreased body temperature observed in WT mice was linked to the loss of BAT.

To assess the persistence of alcohol’s effect on BAT weight and body temperature, we measured these parameters in mice that were fed alcohol then allowed to ‘recover’ without alcohol administration for one month. Consistent with the data presented above, alcohol-fed mice had a significantly lower BAT weight compared to control animals, which did not return to baseline after the one month recovery period (Fig. 2E). This persistent effect on BAT weight was also reflected in our measures of body temperature. Alcohol-fed mice had a significantly lowered body temperature than control mice, but this parameter did not return to baseline levels after the recovery period (Fig. 2F).

To further study alcohol’s effect on thermoregulation we conducted a standard stress test. As expected, control mice responded to a stressor (handling) by increasing their body temperature; however, when alcohol-fed mice were stressed we did not observe a change in body temperature (Fig. 2G).

### BAT of alcohol-fed mice has reduced lipid content

**Figure 3:**
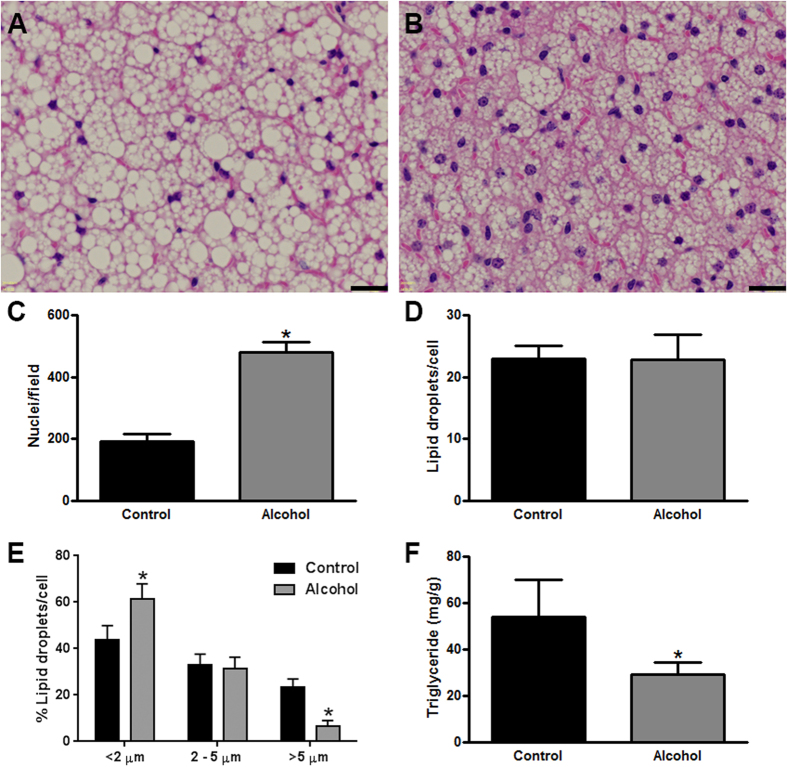
BAT of alcohol-fed mice has reduced lipid content. Representative Hematoxylin and eosin-stained tissue sections of the intrascapular BAT depot from control (**A**) and alcohol consuming mice (**B**). Image analysis of BAT shows a significantly increased number of nuclei/field in alcohol fed mice (**C**). There was no significant difference in the number of lipid droplets/cell in control vs. alcohol-fed mice (**D**); however, alcohol-fed mice had a significantly smaller percentage of large lipid droplets, and a significantly higher percentage of small lipid droplets (**E**). The concentration of triglycerides in the BAT of alcohol-fed was significantly lower (**F**). Scale bars in A and B = 20 μm. (**C–F**) analyzed by Student’s t-test; *p < 0.05. Sample size: (**C–E**), all groups n = 3; (**F**) control and alcohol n = 5.

To gain a better understanding of the alcohol-induced decrease in BAT weight, we conducted a histological examination of this tissue. Hematoxylin and eosin-stained sections of the intrascapular BAT depot revealed a visibly obvious decline in the area occupied by lipid droplets (white spaces) in alcohol-fed mice (Fig. 3A,B). Systematic image analysis of BAT showed that the number of nuclei per field was significantly increased in alcohol-fed mice (Fig. 3C). The number of lipid droplets per cell was not different between control and alcohol-fed mice (Fig. 3D); however, there was a change in the distribution of lipid droplet size (Fig. 3E). Specifically, alcohol-fed mice had significantly more small-sized lipid droplets (diameter <2 μm), and significantly less large-sized lipid droplets (diameter >5 μm). Taken together, this data suggested that the BAT of alcohol-fed mice had become hypotrophic, with the same number of lipid droplets/cell, but these lipid droplets had become smaller. To confirm that there was less lipid in the BAT of alcohol-fed mice, we conducted a biochemical assay of BAT triglyceride content. This assay revealed that the BAT of alcohol-fed mice contained almost half the triglycerides of control mice (Fig. 3F).

### Alcohol increases BAT retinoid content independent of dietary vitamin A content, but dependent on hepatic retinoid stores

**Figure 4:**
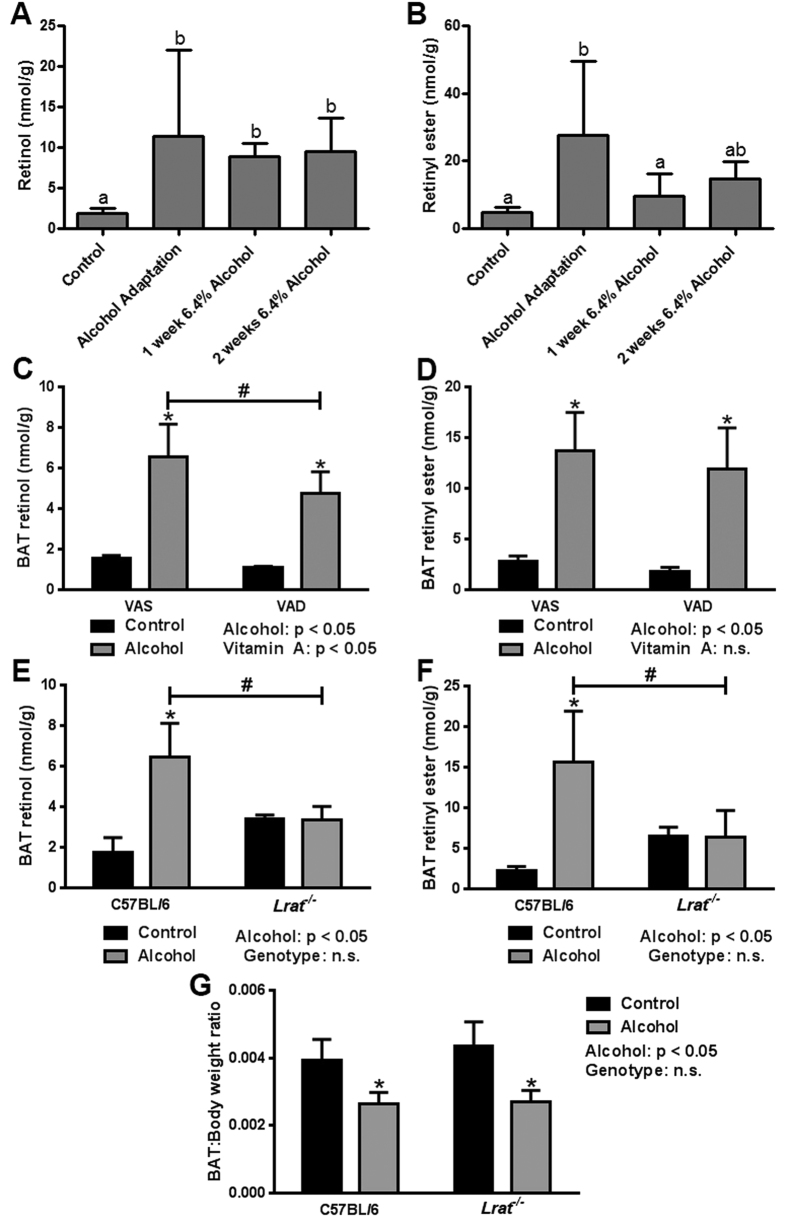
Alcohol increases BAT retinol and retinyl ester content independently of dietary vitamin A content, but dependent on hepatic vitamin A stores. Chronic alcohol consumption was associated with significant increase in BAT concentrations of retinol (**A**) and retinyl ester (**B**). The alcohol-induced increase in BAT retinol and retinyl ester concentration was similar in mice consuming a vitamin A sufficient (VAS) and vitamin A deficient (VAD) diet (**C**,**D**). The alcohol-induced increase in BAT retinol and retinyl ester concentrations was absent in *Lrat*^−/−^ mice (**E**,**F**). The effect of chronic alcohol consumption on BAT mass was retained in *Lrat*^−/−^ mice (**G**). (**A,B**) analyzed by one-way ANOVA; bars with different letters are significantly different; p < 0.05. (**C–G**) analyzed by two-way ANOVA; *p < 0.05 within groups; ^#^p < 0.05 between groups. Sample size: (**A,B**) control n = 12, all alcohol groups n = 6; (**C,D**), VAS control n = 4, VAS alcohol n = 6, VAD control n = 5, VAD alcohol n = 7; (**E–G**) C57BL/6 control n = 5, C57BL/6 alcohol n = 5, *Lrat*^−/−^ control n = 4, *Lrat*^−/−^ alcohol n = 5.

Retinoid signaling has previously been shown to play an important role in BAT function^11^. We have also shown that chronic alcohol consumption has profound effects on tissue retinoid levels, depleting hepatic retinoid levels but driving compensatory increases in other tissues^2^,^3^. To test our working hypothesis that an alcohol-induced disruption of BAT retinoid homeostasis contributed to altered BAT function, we measured BAT retinoid content by HPLC. This analysis revealed that alcohol consumption significantly increased BAT retinol concentration throughout the alcohol-feeding protocol (Fig. 4A), with evidence of significantly elevated levels of retinyl ester as well (Fig. 4B). Two follow-up studies were conducted to determine the origin of the retinoid accumulating in the BAT of alcohol-fed mice. First, to assess the role of dietary retinoid intake, we measured BAT retinoid levels in mice consuming alcohol and a VAD diet. This showed that BAT retinol and retinyl ester levels were significantly increased in alcohol-fed mice consuming either a vitamin A-sufficient (VAS) or -deficient diet (VAD; Fig. 4C,D). There was a small but significant decrease in retinol levels in VAD versus VAS mice consuming alcohol, but no significant difference in the retinyl ester levels between the two diets, suggesting a minor contribution from the diet. Next, we studied *Lrat*^−/−^ mice–which have no hepatic retinoid stores–to determine the contribution of an alcohol-induced redistribution of hepatic retinoid stores to BAT^18^. Strikingly, the alcohol-induced increase in BAT retinol and retinyl ester concentrations observed in WT mice was completely blocked in *Lrat*^−/−^ mice (Fig. 4E,F), suggesting that the major contributor to the increase in BAT retinoid levels in response to alcohol consumption was redistribution from the liver. This finding is consistent with our recently published work on alcohol-induced hepatic retinoid loss^2^.

The above data supported our working hypothesis that an alcohol-induced disruption of BAT retinoid homeostasis contributed to altered BAT function, showing that decreased BAT weight was strongly correlated with increased BAT retinoid content; indeed, even when controlling for the decrease in BAT weight, tissue retinol and retinyl ester levels remain significantly increased in alcohol-fed mice (data not shown). To further test this hypothesis, we measured BAT weight in *Lrat*^−/−^ mice fed alcohol, predicting that by blocking the alcohol-induced increase in BAT retinoid levels we would preserve BAT weight. Our data, however, clearly refuted this prediction, showing that there was no difference in the BAT:body weight ratio of WT versus *Lrat*^−/−^ mice consuming alcohol, with the expected decline in response to alcohol exposure present in both genotypes (Fig. 4G).

### Chronic alcohol consumption is associated with decreased retinoid signaling in BAT, but no change in UCP1 expression

**Figure 5:**
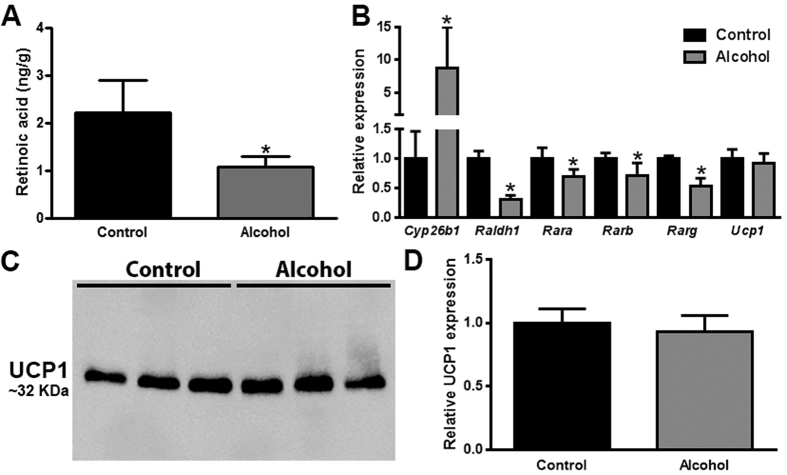
Chronic alcohol consumption is associated with decreased retinoid signaling in BAT, but no change in UCP1 expression. The concentration of retinoid acid in the BAT of alcohol-fed mice is significantly decreased (**A**). The mRNA expression levels of genes involved in retinoid metabolism are significantly dysregulated in the BAT of alcohol consuming mice. The mRNA expression level of UCP1 was unchanged in alcohol-fed mice (**B**), this was confirmed at the protein level as shown by a representative western blot of UCP1 expression in BAT of control and alcohol-fed mice (**C**), with accompanying relative quantification normalized to total protein, as described in the methods (**D**). (**A,B,D**): analyzed by Student’s t-test; *p < 0.05. Sample size: (**A**) control n = 4, alcohol n = 6; (**B**) all groups n = 6; (**C**) all groups n = 3.

Our data from *Lrat*^−/−^ mice showed that BAT retinoid content does not necessarily predict BAT weight in alcohol consuming mice. As introduced, the active metabolite of vitamin A is retinoic acid, whereas retinol and retinyl ester are generally not ascribed any biological activity in cell signaling^3^. Interestingly, although alcohol consumption was associated with increased levels of BAT retinol and retinyl ester, we observed a significant decrease in BAT retinoic acid concentration by more than 50% in alcohol-fed mice (Fig. 5A). To better understand this apparent discrepancy, we measured the expression level of genes involved with tissue retinoid metabolism (Fig. 5B). Our data show that alcohol consumption was associated with significantly decreased levels of *Raldh1*, and significantly increased levels of *Cyp26b1*. The protein product of *Raldh1* catalyzes retinoic acid synthesis, whereas *Cyp26b1* catalyzes retinoic acid breakdown^19^. Thus, the gene transcription signature observed in alcohol-fed mice suggests a state of decreased retinoic acid synthesis and increased retinoic acid breakdown, thereby explaining the observed decreased in BAT retinoic acid levels. Retinoic acid signals through nuclear retinoic acid receptors (RARα, β, and γ)^19^, the expression of which is affected by cellular retinoic acid levels. We found that the expression level of *Rara, Rarb*, and *Rarg* were all significantly decreased in alcohol consuming mice, supporting the notion that retinoic acid signaling was disrupted in the BAT of alcohol-fed mice. As discussed above, UCP1 is an important mediator of heat production in BAT, the expression of which is proposed to be controlled by retinoic acid^11^. Despite the observed decrease in tissue retinoic acid levels and evidence for decreased retinoic acid signaling, we did not observe a significant change in *Ucp1* mRNA expression levels in alcohol-fed mice. This finding was confirmed at the protein level by western blotting (Fig. 5C,D).

## Discussion

Our data show that chronic alcohol consumption is associated with decreased BAT weight in mice, with an accompanying impairment in thermoregulation. An important conclusion drawn from our experimental data is that the alcohol-induced decrease in BAT weight is linked to altered thermoregulation. While alcohol is known to have an acute effect on body temperature, we believe the effects we observed on BAT weight and thermoregulation is a chronic, adaptive effect. This is reflected in the progressive decline in body temperature throughout our alcohol-feeding protocol, as well as the observation that even after a one-month recovery period, the decline in BAT weight and nighttime body temperature persists.

Our data from alcohol-consuming UCP-DTA mice is an important control experiment, which supports our interpretation of the data. If alcohol was affecting thermoregulation independently of its chronic effect on BAT (e.g. by a direct acute effect, or through a shift in circadian rhythm), then we would have expected that UCP-DTA mice would have experienced an alcohol-induced decrease in body temperature like control mice; however, we did not observe this. Alcohol-fed UCP-DTA mice maintained their body temperature, albeit at a lower baseline level, supporting the link between the alcohol-induced decrease in BAT weight and thermoregulation. While we have not definitively ruled out other explanations to explain alcohol’s effects on thermoregulation, our data support the conclusion that the alcohol-induced decrease in BAT weight is linked to altered thermoregulation. Indeed, the notion that mice with reduced BAT mass have an impaired thermogenic capacity is consistent with previous observations from UCP-DTA mice^20^. These animals have a well-described *downshift* in their core body temperature, which averages ~0.9 °C lower than control^20^. The magnitude of this decrease is comparable with our observations from UCP-DTA mice, which were also ~0.9 °C lower than control. Strikingly, alcohol also decreased core body temperature to a similar extent (~1.4 °C lower), indicating that alcohol’s effect on BAT and core body temperature is comparable to its genetically-driven ablation in UCP-DTA mice.

We propose that the primary reason BAT weight is reduced in alcohol-fed mice is the observed decrease in lipid (triglyceride) content. We believe there are two possible mechanisms that could explain this effect. First, BAT lipid content could be decreased because this tissue is experiencing increased activation, which is driving the consumption of its lipid stores via uncoupling of mitochondrial fatty acid oxidation. While the ability of alcohol to increase BAT activity has been previously reported, this conclusion was only inferred from presumed differences in energy expenditure, based on caloric intake and body weight data^21^. Further, we would expect that increased BAT activity would increase the body temperature of alcohol consuming mice, which is contrary to our observations. Alternatively, alcohol may be having a direct effect on lipid metabolism in BAT, whereby alcohol impairs fatty acyl metabolism in BAT, with subsequent reductions in triglyceride levels. As discussed below, the notion that alcohol interferes with BAT lipid metabolism may be a consequence of altered retinoic acid signaling in this tissue. If correct, this would provide a link between our observations of altered lipid *and* retinoid metabolism in the BAT of alcohol consuming mice. Regardless, the data presented in the current manuscript cannot definitively separate these possible mechanisms. Our on-going research is focused on alcohol’s effect on markers of BAT activation, retinoic acid signaling, and lipid metabolism.

We originally hypothesized that alcohol may act in BAT by disrupting retinoic acid signaling, with consequent effects on UCP1 expression and the ability of BAT to generate heat. Despite reports suggesting that retinoic acid can control UCP1 expression^11^, we did not observe any change in the level of UCP1 in the BAT of alcohol-fed mice, even though the concentration of retinoic acid was significantly lower. Interestingly, it has also been reported that BAT UCP1 expression is unchanged in *Raldh1*^−/−^ mice, suggesting that other transcriptional drivers of UCP1 expression may be more important than retinoic acid under certain physiological circumstances^22^. Our current working hypothesis focuses on the interaction between retinoic acid signaling and lipid metabolism in BAT. As recently reviewed by Bonet *et al*., there is a significant literature that links retinoic acid signaling and lipid metabolism in different tissues of the body, including skeletal muscle, liver and adipose^11^. These effects seem to be primarily mediated by the transcriptional regulation of retinoic acid on genes important in lipid metabolism. This is true for genes involved in the major pathways of cellular fatty acid metabolism, including de novo lipogenesis (e.g. SCD1), mitochondrial β-oxidation (e.g. MCAD), and lipolysis (e.g. HSL)^11^. Future studies in our laboratory will focus on alcohol’s effect on lipid metabolism in BAT, and potential interactions with retinoid homeostasis in this tissue. The strong linkage between retinoid signaling, lipid metabolism in BAT, and thermogenesis was underscored in a recent study, which showed that Lipocalin 2 deficient mice have impaired retinoid homeostasis in their adipose tissue, with consequent effects on thermogenesis^23^.

Although we are still exploring the link between alcohol-induced alterations in BAT retinoic acid signaling and impaired thermogenesis, our data clearly demonstrate that alcohol has a profound effect on retinoid homeostasis in BAT. We show that chronic alcohol consumption is associated with altered BAT retinoid levels (including retinoic acid, retinol and retinyl ester), with corresponding changes in the expression level of genes important in retinoid metabolism and signaling. While our previous work has focused on alcohol’s effect on retinoic acid signaling in the liver, the current study shows that alcohol can impair retinoic acid signaling in multiple tissues, supporting the notion that this is a general mechanism of alcohol-induced toxicity, as suggested by others^24^.

All our alcohol-feeding studies employed pair-fed control mice. This experimental approach was taken to ensure isocaloric nutrient intake between experimental groups, but it is important to recognize that pair-fed control mice are metabolically different than mice fed an ad-lib control diet, which may have undetermined effects on BAT physiology. Nevertheless, our conclusions remain valid within the experimental context of the study.

Alcohol abuse has wide-ranging effects on the human body, and contributes to a significant global disease burden^25^; however, the effect of alcohol on BAT function has not been rigorously studied in humans. We are not aware of any human alcohol exposure studies taking advantage of FDG-PET/CT (18F-fluorodeoxyglucose position emission tomography/computed tomography) imaging of BAT, a method that triggered the current research interest in studying BAT physiology in humans^5^. The significance of alcohol’s potential effect on BAT in humans is two-fold. First, alcohol intoxication can put individuals at risk for accidental hypothermia, with alcohol abuse being the most frequently reported factor contributing to hypothermia, particularly in urban settings^26^,^27^,^28^,^29^,^30^. The hypothermic effect of alcohol has multiple mechanisms, including decreased perception of cold, increased peripheral heat loss, and decreased capacity to produce heat^31^. We speculate that alcohol’s effect on BAT is an underappreciated contributor to alcohol’s hypothermic effect, requiring further investigation. The second important implication of our work intersects with the widespread interest in modulating BAT function in the fight against obesity. It is currently thought that interventions that can increase BAT function, will lead to increased energy consumption by BAT, thereby decreasing body weight^32^. Using the reverse logic, if alcohol consumption impairs BAT function, could it therefore be contributing to decreased energy expenditure and the obesity epidemic? The majority of overweight and obese individuals regularly consume alcohol^33^, and the extensive epidemiological literature provides evidence for both a positive and a negative effect of alcohol consumption on body weight^34^. As the obesity epidemic continues to grow, it is increasingly important to determine the impact of even moderate alcohol intake on energy balance and weight gain. In this regard, we show that even with relatively low levels of alcohol consumption, we observe decreased BAT mass and altered thermoregulation. With this in mind, we believe BAT function should be rigorously studied in alcohol-consuming humans, both acutely and chronically.

In summary, mice chronically consuming alcohol have decreased BAT weight and impaired thermoregulation. We believe alcohol has an underappreciated impact on BAT function, which has implications for human health both acutely, in terms of thermoregulation, and chronically, in terms of energy homeostasis. Mechanistically, alcohol’s effects are linked with decreased lipid content in BAT, and altered retinoic acid signaling.

## Methods

### Animals and alcohol feeding protocol

All studies were conducted in age-matched, male, C57BL/6 mice, unless otherwise stated. All animals were housed in the Association for Assessment and Accreditation of Laboratory Animal Care-accredited mouse facility at the Columbia University Medical Center. All studies were approved by the Columbia University Institutional Animal Care and Use Committee, and performed in accordance with the relevant guidelines. Mice were housed in a climate-controlled barrier facility with a 12-hour light-dark cycle. The majority of experiments were conducted using a vitamin A-sufficient (VAS; 4 IU/g diet), high-fat formulation of the Lieber-DeCarli liquid diet (Bio-Serv, Flemington, NJ)^35^. Follow-up studies utilized different formulations of the Lieber-DeCarli liquid diet, including a vitamin A-deficient (VAD; 0 IU/g diet), high-fat formulation, as well as a VAS, low-fat formulation (all manufactured by Bio-Serv). A detailed description of our alcohol-feeding protocol has been published elsewhere^3^. In brief, to adapt mice to the liquid diets, we used an alcohol adaptation period consisting of one week 0% alcohol, one week 2.1% alcohol, and one week 4.2% alcohol. Following this period, mice were fed 6.4% alcohol for up to 4 weeks. Control mice were pair-fed alcohol-free liquid diets, being provided with the average volume of liquid diet that alcohol-fed mice had consumed during the previous 48 h. Mice in the ‘recovery’ experiment were fed alcohol throughout the alcohol adaptation period, and then provided with an alcohol-free diet for 1 month, as previously described^3^. Peak blood alcohol levels achieved using this alcohol feeding protocol have previously been reported by our group, and are in excess of 0.01%^36^. The enzyme Lecithin:retinol acyltransferase (LRAT) catalyzes the synthesis of retinyl ester from retinol^37^. Follow-up alcohol-feeding studies in *Lrat*^−/−^ mice in a congenic C57BL/6 background compared with WT C57BL/6 mice,were performed. The genotype of *Lrat*^−/−^ mice was confirmed genetically by PCR, and phenotypically by the absence of hepatic retinoid stores (data not shown)^18^. Follow-up studies into the relationship between reduced BAT weight and altered thermogenesis were performed in UCP-DTA mice, which have targeted ablation of their BAT achieved by driving diphtheria toxin A (DTA) expression under the control of the UCP1 promoter, as previously described^17^. At the end of our alcohol feeding studies, mice were humanely euthanized and tissues were collected and immediately snap frozen in liquid nitrogen or fixed for histology. All data presented for BAT was collected from the intrascapular BAT depot. This tissue was initially removed, and then the surrounding WAT was carefully dissected away. The data presented for WAT was collected from the epididymal visceral adipose tissue depot.

### Measurement of core body temperature

The core body temperature of experimental mice was measured using a rectal probe connected to a digital thermometer (Braintree Scientific, Braintree, MA). Measurements were taken at noon and midnight on different days throughout the alcohol-feeding protocol. In order to obtain an accurate reading of core body temperature, mice were habituated to this procedure and care was taken not to stress the mice prior to temperature measurement. It is known that handling stress can increase murine core body temperature^38^. To determine the effect of alcohol on this stress response, we measured core body temperature in mice at baseline and 20 minutes after handling.

### BAT histology and image analysis

Dissected BAT was fixed overnight in 10% formalin, and processed for Hematoxylin and eosin staining by the Columbia University Medical Centre’s Molecular Pathology core facility. Stained slides were imaged using an FSX100 microscope (Olympus, Tokyo, Japan). Image analysis was performed using the *count* and *measure* functions available on the proprietary FSX100 software (Olympus).

### Analysis of BAT lipid content

BAT retinol and retinyl ester concentrations were measured using HPLC, according to previously published protocols^3^,^39^. In brief, lipids were extracted from tissue homogenates using hexane and analyzed using a Waters modular HPLC system (Waters, Milford, MA). Chromatographic separation of extracted lipids was achieved using a Waters Symmetry C18 column (4.6 × 250 mm), and retinol and retinyl esters were measured at a peak absorbance of 325 nm using a photodiode array (Waters). Quantification of these lipids was calculated based on the recovery of a retinyl acetate internal standard. Tissue concentrations of retinoic acid were measured by LC/MS/MS, using a Xevo TQ MS Acquity UPLC system (Waters), as previously described^40^. Note, in all cases, when we refer to retinol, retinyl ester and retinoic acid, we are referring to the all-*trans*-isomers of these compounds. BAT triglyceride content was measured in lipids extracted using a standard Folch solution (2:1 methanol chloroform)^41^, and measured using a liquid triglycerides reagent (Thermo Fisher Scientific, Waltham, MA).

### BAT gene expression analyses

Standard protocols, as previously described, were followed for RNA extraction, cDNA synthesis and qPCR^36^. Gene (mRNA) expression levels of *Cyp26b1, Raldh1, Rara, Rarb, Rarg* and *Ucp1* were compared relative to the reference gene *18s.* The primer sequences used to amplify our cDNA are provided in Table 1.

### UCP1 protein expression analysis

The expression level of UCP1 was determined in protein homogenates of BAT prepared from control and alcohol-fed mice. In brief, total protein was separated by SDS-PAGE (12% acrylamide) and transferred to a nitrocellulose membrane, using standard protocols. UCP1 expression was determined using an anti-UCP1 rabbit polyclonal antibody (1:5,000 dilution; ab10983; Abcam, Cambridge, UK), which was visualized using an HRP-conjugated anti-rabbit secondary antibody (1:10,000 dilution; NA934V; GE Healthcare Ltd; Chicago IL, USA), and chemiluminescence (SuperSignal West Pico Chemiluminescent Substrate; Thermo Fisher Scientific, Waltham, MA, USA). Total protein was used as a loading control to normalize our analysis of UCP1 expression levels. Total protein was visualized using BioRad’s proprietary Stain-Free chemistry (BioRad, Hercules, CA, USA), utilizing TGX Stain-Free gels and a ChemiDoc Touch Imaging system. UCP1 expression and total protein levels were visualized in the same membrane, and the normalized level of UCP1 expression was calculated using Image Lab (v5.2.1; BioRad).

### Statistical analyses

All data were compiled using Excel (Microsoft, Redmond, WA) and analyzed using GraphPad Prism (GraphPad Software, La Jolla, CA). As specified in the figure legends, statistical testing was performed using a Student’s t-test, one-way ANOVA, or two-way ANOVA, depending on the experimental design. The sample size for each experimental group is also presented in the figure legends. In all cases, a p-value < 0.05 was considered statistically significant.

## Additional Information

**How to cite this article:** Blaner, W. S. *et al*. Chronic alcohol consumption decreases brown adipose tissue mass and disrupts thermoregulation: a possible role for altered retinoid signaling. *Sci. Rep.* **7**, 43474; doi: 10.1038/srep43474 (2017).

**Publisher's note:** Springer Nature remains neutral with regard to jurisdictional claims in published maps and institutional affiliations.

## Figures and Tables

**Table 1:** Primer sequences used for qPCR.

Gene of interest		Primer sequence (5′-3′)	Amplicon size (bp)
Symbol	Full name		
*18 s*	18 s ribosomal RNA	F–CCA TCC AAT CGG TAG TAG CG	100
		R–GTA ACC CGT TGA ACC CCA TT	
*Cyp26b1*	Cytochrome P450, family 26, subfamily b, polypeptide 1	F–GCA GTA TAT GCT TAT GAC ATC TGA ATC	77
		R–CCT GAC CAC TCA CCA ACA AA	
*Raldh1*	Retinal dehydrogenase 1	F–TGG GAA TAC CGT GGT TGT CAA GC	125
		R–TTG GCC CAT AAC CAG GGA CAA T	
*Rara*	Retinoic acid receptor, alpha	F–CAC GCC TGAG CAA GAC ACA ATG A	106
		R–CTA GCT CCG CTG TCA TCT CAT AG	
*Rarb*	Retinoic acid receptor, beta	F–GGG CAT GTC CAA AGA GTC TGT TAG	101
		R–CTA GCT CCG CTG TCA TCT CAT AG	
*Rarg*	Retinoic acid receptor, gamma	F–ACT AAG GGA GCA GAA AGG GCT AT	112
		R–TCG AGG AGT CGT CCT CAA ACA	
*Ucp1*	Uncoupling protein 1	F–GGC AGC CTA CAG AGG TCG	117
		R–AGC TTT CTG TGG TGG CTA TAA CT	
